# Influencing factors of bone mass abnormalities among postmenopausal women in Tibet, China

**DOI:** 10.1186/s12889-023-17015-6

**Published:** 2023-10-25

**Authors:** Huaichang Zhong, Yaxi Zhou, Peng Wang, Qundi Jia, Yang Wan, Hai Xiong

**Affiliations:** 1 West China School of Public Health and West China Fourth Hospital, Sichuan University, Chengdu, Sichuan China; 2 Tibet University Medical college, Lhasa, Tibet China

**Keywords:** Tibet, Postmenopausal women, Bone mass abnormalities, Influencing factors

## Abstract

**Background:**

This study aimed to provide a reference for the prevention and treatment of abnormal bone mass in postmenopausal women by analysing the current situation and influencing factors of bone mass abnormalities in Tibet.

**Methods:**

A total of 229 postmenopausal Tibetan women were randomly selected from six counties by a multistage cluster random sampling method. Multiple logistic regression was utilized to analyse the status and influencing factors of bone mass abnormalities in postmenopausal Tibetan women.

**Results:**

Among 229 postmenopausal Tibetan women, the prevalence of osteopenia and osteoporosis was 54.6% and 9.6%, respectively. Age {odds ratio (OR) = 0.022 [95% confidence interval (CI) = 0.003 ~ 0.163]}, BMI [OR = 441.902 (20.899,9343.717)], altitude [OR = 18.818 (1.391,254.585)], and creatinine (CREA) levels [OR = 0.895 (0.825 ~ 0.971)] were significantly associated with the risk of osteoporosis.

**Conclusion:**

Postmenopausal Tibetan women had high rates of abnormal bone mass. Age, BMI, altitude and CREA levels were associated with osteoporosis. It is suggested that relevant departments should take targeted measures to promote health education on the prevention of osteoporosis in the general population and increase the screening of high-risk groups for osteoporosis to improve the bone health of postmenopausal Tibetan women.

## Background

Osteoporosis (OP) is a systemic skeletal disorder characterized by low bone mass, microarchitectural deterioration in bone tissue and enhanced bone fragility, leading to increased fracture risk, disability and mortality [1]. Bone mass abnormalities include osteopenia and osteoporosis. Osteopenia, defined by the World Health Organization as a bone mineral density (BMD) T score between − 2.5 and − 1.0 [2], implies an increased risk of fracture in diagnosed individuals compared to healthy individuals but is not severe enough to be considered a disease state. Osteoporosis is highly prevalent in postmenopausal women due to their menopausal state and reduced oestrogen levels. It is estimated that 40% of postmenopausal women have osteoporosis, and 50% of postmenopausal women may have an osteoporotic fracture in their lifetime [3].

Currently, studies suggest that race is an important influencing factor for osteoporosis. In the United States, the prevalence of osteoporosis among Mexican Americans is more than twice that among non-Hispanic black adults [4]. Moreover, in Canada, BMD values were higher in individuals with Chinese race/ethnicity than in white Canadians [5].

Furthermore, factors of geographical environments, such as altitude, atmospheric oxygen content, and the duration of sun exposure, may also impact human bone mass status [6, 7]. At present, there are a few studies about the influence of high altitude on human bone mass [8, 9]. However, most studies have focused on the change in bone mass status in people who migrate from low-altitude areas to high-altitude areas for a short period of time, and there is a lack of studies on populations who have lived in plateaus for generations.

The Tibet Autonomous Region is located in southwest China, has an average altitude of over 4,000 m, and has a unique topography, natural climate and traditional culture. The large range of altitudes in Tibet provides greater representativeness to study the relationship between altitude and osteoporosis. Tibetan individuals are an ethnic minority group in China with a total population of over 7 million, mainly distributed in Tibet. Owing to the influence of the geographical environment and customs, Tibetans have particular behaviours and lifestyles.

This was a population-based study to explore the influencing factors of bone mass abnormalities in postmenopausal Tibetan women and analyse the effect of different altitudes on bone mass status to provide a reference for corresponding prevention and treatment efforts.

## Methods

### Data source and study participants

This cross-sectional study population was part of the Tibetan Chronic and Endemic Disease Prevention on the Qinghai-Tibet Plateau program. The program was carried out in some parts of Tibet from July to October 2021. The Tibet Autonomous Region consists of six regions, namely, Lhasa, Shannan, Naqu, Linzhi, Rikaze, Changdu and Ali. The program adopted a multistage cluster random sampling strategy. In stage 1, we randomly selected 3 of the 6 regions. In stage 2, we randomly selected 1 to 3 counties from each region to obtain a total of six counties. In stage 3, we randomly selected 2 townships from each county. In stage 4, we randomly selected 2 to 3 urban residential communities or rural village communities from each township. In stage 5, we used a cluster sampling method to include all residents (aged > 15 years) in the community in our program. Residents were asked in advance to participate in questionnaires and health checks at the medical points set up in the communities. Tibetans (aged > 15 years) were selected for a face-to-face survey.

All residents who participated in our program signed an informed consent form after receiving information about the content of the project (participants with educational experience signed the form, while those with no educational experience were informed of the content of the project and provided consent by fingerprinting). A total of 1761 residents were ultimately surveyed, among whom 239 were postmenopausal women.

The number of postmenopausal women in the program could support the calculated sample size of this study. The sample size was calculated as follows using the prevalence of osteoporosis:

$$\mathrm{N}=\frac{{\mathrm{Z}}_{1-\mathrm{\alpha }/2}^{2}\mathrm{P}\left(1-\mathrm{P}\right)}{{\mathrm{d}}^{2}}$$

According to previous research [10], the estimated p value of the prevalence of osteoporosis in this study was 0.206. The value of α was 0.05 (two-sided), the value of Z_1-a/2_ was 1.96, and the value of d was 0.05. According to the formula, the sample size was 224 people. Considering factors such as refusal to visit and missing data in the actual survey, an additional 5% was added on the basis of the calculated sample size, so the final sample size was expected to be 236.

The inclusion criteria were as follows: ① postmenopausal Tibetan women (one or more complete years since the cessation of their menstrual cycle); and ② women with complete data on biochemical indicators and bone mineral density testing.

The exclusion criteria were as follows: ① participants diagnosed with cardiovascular disorders, cerebrovascular pathologies, diabetes, chronic arthritis, liver disorders, hyperthyroidism, and hyperparathyroidism; ② participants who had recently taken drugs affecting bone metabolism; and ③ participants with missing data on covariates. A total of 229 patients were ultimately included in this survey.

### BMD and blood biochemistry testing

All measurements were performed by trained investigators using calibrated equipment following the manufacturer's instructions.

The BMD of the left distal forearm was measured by a dual-energy X-ray absorptiometry densitometer (DXA, AKDX-09 W-I, Shenzhen Xray Electric Co., Shenzhen, China), which was based on the collection of 12,159 forearm radial bone mineral density values from all over China to build a database, according to the protocol (precision error; ≤ 1.0% CV in vivo). The DXA instrument was taken to study sites by vehicle during the survey. Quality control processes were carried out according to the manufacturer's operating manual.

Fasting venous blood was collected from the participants using a 5-ml vacuum coagulant tube and centrifuged after 2 h at room temperature, and serum was collected for testing. The participants’ blood biochemistry indicators, including uric acid (UA), blood creatinine (CREA), total cholesterol (TC), triglyceride (TG), low-density lipoprotein cholesterol (LDL-C), and high-density lipoprotein cholesterol (HDL-C) levels, were measured by the Tibet Autonomous Region Institute of Tibetan Medicine with a fully automated biochemical analyser (Hitachi 7600, Hitachi, Tokyo, Japan).

### Diagnostic criteria for abnormal bone mass

Osteoporosis and osteopenia were defined according to World Health Organization criteria [11]. The BMD T score was the number of standard deviations (SDs) that compared the BMD value to a reference mean for young adults of the same sex. T scores were calculated as follows: (measured BMD value—peak BMD)/SD. Women were characterized as having osteoporosis when the T score was equal to or less than -2.5 SD, osteopenia when the T score was between -1.0 SD and -2.5 SD and normal bone mass when the T score was equal to or greater than − 1.0 SD.

### Sociodemographic characteristics, lifestyle characteristics and BMI

The sociodemographic characteristics included age (1 = 40 ~ 54, 2 = 55 ~ 64, 3 = 65 ~), registered permanent residence (1 = agricultural, 2 = urban) and education level (1 = no school, 2 = primary school, 3 = junior high school and above). Lifestyle characteristics included smoking status (1 = smoker, 2 = ever smoker, 3 = nonsmoker), drinking status (1 = drinker, 2 = ever drinker, 3 = nondrinker) and physical exercise, assessed by frequency (1 = never, 2 = less than once per week, 3 = once or twice per week, 4 = three times per week or more). Body mass index (BMI) was calculated using the following equation: weight (kg)/[height (m)]^2^ (1: BMI < 18.5 kg/m^2^, 2: 18.5 kg/m^2^ ≤ BMI < 24 kg/m^2^, 3: BMI ≥ 24 kg/m^2^).

### Statistical analysis

Data were collected by Epidata 3.1 software, and statistical analysis was performed by SPSS 25.0 software (IBM, Armonk, New York, USA). Quantitative variables are described as the mean ± standard deviation. One-way analysis of variance and LSD t test were used for comparison of quantitative data between groups and further pairwise comparison, respectively. Categorical variables are described by the percentage or constituent ratio n (%), and the chi-square test was used for comparison of categorical variables between groups.

Prior to conducting data analysis, multivariate outliers were assessed by using Mahalanobis distance. Items with a Mahalanobis distance of *p* < 0.001 were considered multivariate outliers. No significant outliers were detected.

To determine the independent effect of variables related to osteoporosis in postmenopausal women in Tibet, multivariate regression analysis was performed to adjust for confounding factors, including age, BMI, registered permanent residence, education level, physical activity, smoking and drinking status, high-altitude heart disease, hypertensive heart disease, history of fracture, and residence altitude. The level of statistical significance was set at 0.05 for all analyses.

## Results

The general characteristics of the subjects enrolled in this study are shown in Table 1. Of the 229 postmenopausal women, 82 (35.8%) had normal bone mineral density, 125 (54.6%) had osteopenia, and 22 (9.6%) had osteoporosis. The results indicated that registered permanent residence, education level, and physical exercise were not significantly different among groups. Osteoporosis was significantly more prevalent among age, BMI, the hypertensive heart disease, and high-altitude groups (*P* < 0.05).

**Table 1:** General characteristics of the respondents

Variable	Women with Normal Bone Mass (*n* = 82)	Women with Osteopenia (*n* = 125)	Women with Osteoporosis (*n* = 22)	χ^2^	*P* value
Age (years)				42.364	< 0.001
40 ~ 54	38	28	4		
55 ~ 64	36	54	2		
≥ 65	8	43	16		
BMI (kg/m^2^)				44.572	< 0.001
< 18.5	1	9	5		
18.5 ~ 23.9	25	78	14		
≥ 24.0	56	38	3		
**Registered permanent residence**				1.846	0.397
agricultural	75	113	18		
urban	7	12	4		
**Education level**				4.619	0.329
No school	70	105	20		
primary school	10	20	2		
junior high school and above	2	0	0		
**physical exercise**				6.843	0.336
never	62	86	13		
less than once per week	4	13	2		
once or twice per week	4	6	0		
three times per week or more	12	20	7		
**Smoking status**				4.253	0.373
smoker	0	4	0		
ex-smoker	0	1	0		
nonsmoker	82	120	22		
**Drinking status**				2.266	0.687
drinker	1	4	0		
ex-drinker	2	4	0		
nondrinker	79	117	22		
**High-Altitude Heart Disease**				0.008	0.996
yes	7	11	2		
no	75	114	20		
**Hypertensive heart disease**				12.098	0.002
yes	2	1	3		
no	80	124	19		
**History of fractures**				0.727	0.695
yes	4	6	2		
no	78	119	20		
**Altitude**				11.007	0.026
≤ 3500	36	80	15		
3501 ~ 4700	20	19	5		
> 4700	26	26	2		

The biochemical indicators of the subjects are presented in Table 2. The results of one-way ANOVA showed statistically significant differences in TC, LDL-C, and CREA levels between the normal bone mass group and the osteoporosis group and between the osteopenia group and the osteoporosis group (*P* < 0.05), while there were no significant differences between the normal bone mass group and the osteopenia group.

**Table 2:** Different biochemical indicators of the respondents

Variable	Women with Normal Bone Mass (*n* = 82)	Women with Osteopenia (*n* = 125)	Women with Osteoporosis (*n* = 22)	P	*P*^1^	*P*^2^	*P*^3^
TC	6.20 ± 1.57	6.22 ± 1.69	5.22 ± 1.02	0.023	0.925	0.011	0.007
TGs	1.33 ± 0.83	1.38 ± 0.85	1.22 ± 0.71	0.691	0.673	0.583	0.407
HDL-C	1.63 ± 0.47	1.60 ± 0.48	1.62 ± 0.70	0.880	0.616	0.899	0.860
LDL-C	3.05 ± 0.94	3.01 ± 0.99	2.38 ± 0.62	0.010	0.803	0.004	0.004
HCY	20.99 ± 5.30	20.42 ± 5.77	19.06 ± 5.13	0.341	0.465	0.148	0.291
UREA	7.61 ± 2.55	7.31 ± 2.46	6.30 ± 2.69	0.096	0.396	0.031	0.084
CREA	56.11 ± 16.49	54.56 ± 23.39	43.29 ± 11.00	0.030	0.589	0.009	0.017
UA	339.58 ± 96.40	335.43 ± 95.87	281.59 ± 70.47	0.028	0.569	0.080	0.017

p: Value of one-way ANOVA

P^1^: Normal bone mass group compared with the osteopenia group

p^2^: Normal bone mass group compared with the osteoporosis group

p^3^: Osteopenia group compared with the osteoporosis group

### Multifactor analysis of factors influencing abnormal bone mass in postmenopausal Tibetan women

The multivariate logistic regression analysis of osteoporosis-related factors in postmenopausal women is displayed in Table 3. Age { OR = 0.022 [95% confidence interval (CI) = 0.003 ~ 0.163]}, BMI [OR = 441.902 (20.899,9343.717)], altitude [OR = 18.818 (1.391,254.585)], and CREA levels [OR = 0.895 (0.825 ~ 0.971)] were significantly associated with the risk of osteoporosis.

**Table 3:** Multiple logistic regression analysis of abnormal bone mass in the respondents

	**variable**	***β***	**Standard error**	***Wald***	***P* value**	***OR (95% CI)***
**Women with Osteopenia**	Age (years)					
	40 ~ 54	-1.997	0.506	15.579	< 0.001	0.136 (0.050 ~ 0.366)
	55 ~ 64	-1.344	0.473	8.058	0.005	0.261 (0.103 ~ 0.660)
	BMI (kg/m^2^)					
	< 18.5	2.624	1.104	5.645	0.018	13.792 (1.583 ~ 120.162)
	18.5 ~ 23.9	1.600	0.371	18.626	< 0.001	4.955 (2.396 ~ 10.250)
	Altitude					
	≤ 3500	0.249	0.413	0.363	0.547	1.283 (0.571 ~ 2.883)
	3501 ~ 4700	-0.265	0.499	0.283	0.595	0.767 (0.289 ~ 2.038)
	CREA	-0.010	0.009	1.409	0.235	0.990 (0.973 ~ 1.007)
	UA	< 0.001	0.002	0.041	0.840	1.000 (0.996 ~ 1.005)
	TC	-0.194	0.318	0.370	0.543	0.824 (0.441 ~ 1.538)
	LDL-C	0.397	0.525	0.571	0.450	1.487 (0.531 ~ 4.160)
	Hypertensive heart disease	-0.224	1.286	0.030	0.862	0.799 (0.064 ~ 9.933)
**Women with Osteoporosis**	Age (years)					
	40 ~ 54	-3.797	1.011	14.107	< 0.001	0.022 (0.003 ~ 0.163)
	55 ~ 64	-4.339	1.015	18.295	< 0.001	0.013 (0.002 ~ 0.095)
	BMI (kg/m^2^)					
	< 18.5	6.091	1.557	15.307	< 0.001	441.902 (20.899 ~ 9343.717)
	18.5 ~ 23.9	2.318	0.844	7.548	0.006	10.159 (1.943 ~ 53.107)
	Altitude					
	≤ 3500	1.051	1.248	0.709	0.400	2.861 (0.248 ~ 33.040)
	3501 ~ 4700	2.935	1.329	4.876	0.027	18.818 (1.391 ~ 254.585)
	CREA	-0.111	0.042	7.073	0.008	0.895 (0.825 ~ 0.971)
	UA	-0.006	0.006	1.126	0.289	0.994 (0.983 ~ 1.005)
	TC	1.243	0.761	2.668	0.102	3.467 (0.780 ~ 15.412)
	LDL-C	-2.444	1.268	3.717	0.054	0.087 (0.007 ~ 1.042)
	Hypertensive heart disease	4.798	2.610	3.380	0.066	121.286 (0.728 ~ 20,205.958)

Control group: Women with normal bone mass

## Discussion

The rapid decline in oestrogen levels due to menopause affects the body's immune, secretion and regulatory functions, which in turn causes the activation and proliferation of T cells, ultimately resulting in a significant reduction in bone mass and osteoporosis. With no effective way to eradicate osteoporosis, the prevention of osteoporosis is particularly important [12].

Among the 229 postmenopausal Tibetan women in this study, 82 (35.8%) had normal bone mineral density, 125 (54.6%) had osteopenia and 22 (9.6%) had osteoporosis; that is, the majority of postmenopausal women (64.2%) were still in a state of abnormal bone mass.

Multivariate regression analysis showed that older age and a low BMI were risk factors for osteoporosis. After menopause, oestrogen deficiency leads to the destruction of the balance between osteoblasts and osteoclasts, and bone loss is further aggravated with increasing age [13]. High BMI is a protective factor against osteoporosis, which increases the mechanical loads and strains associated with obesity, prevents age-related bone loss, and increases peak bone mass, favourably affecting the skeleton [14, 15].

Altitude plays an important role in affecting bone mass. Some relevant studies [16, 17] have indicated that high altitude is a risk factor for osteoporosis, which could be attributed to the low-oxygen environment affecting the natural metabolism of bone tissues, disrupting the balance between bone resorption and bone formation and ultimately leading to osteoporosis. However, to a certain extent, the Tibetan population, as an ethnic group that has lived on the plateau for generations, can adapt to the hypoxic environment of the plateau better than people who migrate from low- to high-altitude environments for a short period of time by regularizing themselves. Previous studies demonstrated that Tibetans living at higher altitudes tended to have lower haemoglobin concentrations and higher respiratory rates, carbon monoxide levels, and blood flow velocities than those living at lower altitudes, all of which are favourable for tolerance to hypoxic environments [18, 19]. In addition, some animal studies have observed that high altitude has a positive effect on promoting bone development and enhancing bone density. For example, sheep with high alpine grazing had higher bone mineral content and density than sheep grazing in lowlands [20], and high altitude had positive effects on the tibial growth plates in poultry [21]. The findings of this research indicated that the prevalence of osteopenia and osteoporosis was lower in high-altitude areas, which was consistent with the conclusion of Luo DJ [22]. On the one hand, at high altitudes in Tibet, sunlight and ultraviolet intensity are sufficient to produce enough vitamin D to help with the absorption of calcium in the body [23]; on the other hand, Tibet is involved in animal husbandry and has an abundance of dairy products. The demand for dairy products is greater among residents of high-altitude areas due to their high calorie content; simultaneously, dairy products also promote bone density and prevent the loss of bone calcium because of their calcium-rich content [24]. In summary, the mechanism of the effect of altitude on populations living in highlands for generations needs further study.

Our research showed that the creatinine level in the osteoporosis group was lower than that in the other groups, suggesting the possibility of abnormal renal function, which can contribute to calcium metabolism disorders, leading to a large amount of calcium ions being excreted through the urine and ultimately resulting in osteoporosis [25]. In addition, low creatinine levels might also be associated with inadequate nutritional intake, which is detrimental to the enhancement of bone density [26].

Hypertensive heart disease encompasses anatomical changes and physiological alterations in the myocardium, coronary arteries, and great vessels, which is an important underlying mechanism of the morbidity and mortality of individuals with blood pressure-related cardiovascular disease [27]. Numerous studies have demonstrated a strong association between cardiovascular disease and osteoporosis [28, 29]. In this study, univariate analysis showed that hypertensive heart disease was associated with osteoporosis. The effects of hypertension on bone mineral density are multifaceted. On the one hand, hypertension can lead to the elevation of parathyroid hormone levels, which contributes to higher blood calcium levels, resulting in lower bone calcium content [30]; on the other hand, sympathetic excitation in hypertensive patients interferes with the natural process of bone resorption and bone formation, consequently decreasing bone density [31]. Diastolic function of the heart and bone density are also strongly correlated. First, calcium ions need to enter the circulation to form blood calcium, which is then deposited in the skeleton to increase bone density; this process is impaired by diastolic dysfunction of the heart, resulting in circulation abnormalities. Next, people with severe diastolic function abnormalities may experience discomfort, thereby reducing their physical activity, which leads to diminished calcium absorption. As a final note, cardiac failure also affects kidney function, causing a decrease in calcium ions [32].

There were some limitations in this research. First, the sample size included in this study was limited, which might result in some bias in the findings. Second, this cross-sectional study was insufficient to make causal inferences, and thus, the conclusions need to be further verified. Third, information on the volunteers' disease status was obtained in the form of questionnaires, which might lead to bias, as some people could be unsure as to whether they had the disease or not. Fourth, there were some confounding factors such as years since menopause, number of births and etc. that were not included in this study, which might produce some bias in the results.

## Conclusion

Postmenopausal Tibetan women had high rates of abnormal bone mass. Age, BMI, altitude and CREA levels were associated with osteoporosis. It is suggested that relevant departments should take targeted measures to promote health education on the prevention of osteoporosis in the general population and increase the screening of high-risk groups of osteoporosis to improve the bone health of postmenopausal Tibetan women.

## Data Availability

The datasets generated and/or analysed during the current study are available from the corresponding author upon reasonable request.

## Abbreviations

OP: Osteoporosis
PMOP: Postmenopausal osteoporosis
BMI: Body mass index
BMD: Bone mineral density
UA: Uric acid
CREA: Blood creatinine
TC: Total cholesterol
TG: Triglyceride
LDL-C: Low-density lipoprotein cholesterol
HDL-C: High-density lipoprotein cholesterol
